# Postoperative Pain and Clinical Outcome Following Two- and Three-Port Video-Assisted Thoracoscopic Surgery for Secondary Spontaneous Pneumothorax

**DOI:** 10.3390/jcm11051404

**Published:** 2022-03-04

**Authors:** Stephen Fung, Kefah Jaber, Marius Kivilis, Alexander Rehders, Anja Schauer, Levent Dizdar, Wolfram-Trudo Knoefel

**Affiliations:** Department of Surgery, University Hospital Duesseldorf and Heinrich-Heine-University Duesseldorf, 40225 Duesseldorf, Germany; stephen.fung@med.uni-duesseldorf.de (S.F.); kefah.jaber@med.uni-duesseldorf.de (K.J.); marius.kivilis@med.uni-duesseldorf.de (M.K.); rehders@med.uni-duesseldorf.de (A.R.); anjamaria.schauer@med.uni-duesseldorf.de (A.S.); levent.dizdar@med.uni-duesseldorf.de (L.D.)

**Keywords:** two-port VATS, three-port VATS, postoperative pain, clinical outcome

## Abstract

Background: Two-port (2P) and three-port (3P) video-assisted thoracoscopic surgery (VATS) are well-established surgical methods for the treatment of complicated spontaneous pneumothorax (SP). However, a comparison between both techniques, in terms of clinical outcomes in patients with secondary spontaneous pneumothorax (SSP), is unreported. The aim of this study was to evaluate and compare postoperative pain, as well as clinical outcome, following 2P and 3P VATS for SSP in our institution. Methods: Between January 2008 and December 2020, we retrospectively analyzed the data of 115 SSP patients treated by VATS in our institution. Fifty-two patients underwent 2P-VATS, while 63 patients were treated by 3P-VATS. The total dose of analgesic use per stay (opioid and non-opioid), length of hospital stay (LOS), operation time, total area of pleurectomy, recurrence rates and postoperative complications were compared between both groups. Results: The 3P-VATS group had a significantly higher total dose of analgesic use compared with the 2P-VATS patients. The LOS and mean operation time were significantly shorter in the 2P-VATS group. A larger area of pleurectomy was resected using 3P-VATS compared to 2P-VATS. The postoperative complications and recurrence of SSP during a median follow-up period of 76.5 months were similar in both groups. Conclusion: 2P-VATS is a safe surgical technique. It is associated with a short LOS and less postoperative pain, and, thus, low analgesic use.

## 1. Introduction

Spontaneous pneumothorax (SP) describes the presence of air, without preceding trauma, within the pleural space. SP in patients with an underlying pulmonary disease is defined as secondary spontaneous pneumothorax (SSP). In most cases, chronic obstructive pulmonary disease (COPD) is the etiological cause in patients who are 45 years of age or older [1]. The incidence of SSP has been reported as approximately 2.0 and 6.3 cases per 100,000 individuals per year in females and males, respectively [2]. For complicated SSP (persistent air leak following chest tube treatment, or recurrence), the current guidelines recommend VATS for the surgical treatment of operable cases [1,3,4]. However, high morbidity rates have been reported after surgery for SSP [5,6]. The high rates of morbidity depend not only on the underlying pulmonary disease, but also on the surgical technique used. Regarding the treatment of primary spontaneous pneumothorax (PSP), recent studies have reported low morbidity rates and less postoperative pain when using a low number of access ports for VATS [7,8,9,10,11,12]. Although, nowadays, three-port (3P) and two-port (2P) VATS are well-established surgical techniques for the treatment of complicated SP; a comparison between both techniques, in terms of postoperative outcome following SSP treatment, is unreported. Thus, the aim of this study was to analyze and compare postoperative pain and clinical outcome after 2P-VATS and 3P-VATS for SSP in our institution.

## 2. Materials and Methods

We retrospectively reviewed the data of 115 patients with secondary spontaneous pneumothorax (SSP), treated either by two-port VATS (2P-VATS) or three-port VATS (3P-VATS) between January 2008 and December 2020 in our institution. Fifty-two patients underwent 2P-VATS, while 63 patients were treated with the conventional 3P-VATS. Indications for surgery included persistent air leaks for more than 5 days following chest tube treatment (*n* = 50) and second ipsilateral or first contralateral recurrent pneumothorax (*n* = 65). Prior to surgery, a computed tomography scan of the lungs was performed to detect the cause of the SSP, and to determine the extent of a bullous disease. A team of three specialized thoracic surgeons (WTK, AS and AR) made the indication for surgery. Notably, the indications for surgery were made individually, depending on the comorbidity and underlying pulmonary disease, as well as the patient’s choice. Patients with incomplete follow-up data and patients who underwent other treatment modalities (e.g., thoracotomy, VATS pleural abrasion, observation, needle aspiration and chest tube drainage) were excluded from this study.

Patient clinical and surgical characteristics (Table 1 and Table 2), including age, sex, body mass index (BMI), side of pneumothorax, COPD stage, size of pneumothorax, number of resected lung segments, total area of resected parietal pleura, length of hospital stay (LOS), operation time, postoperative length of air leak, postoperative complications, and total dose of opioid and non-opioid use per stay, were retrieved from medical records.

The pneumothorax size was assessed using the regression formula derived from Collins et al. [13]. The area of the resected pleura was measured in square centimeters (cm^2^), as denoted in the pathology results. The operation time (in minutes) was defined as the time from skin incision to the end of skin closure. A postoperative prolonged air leak was defined as a persistent air leak for more than 5 days after VATS. Postoperative recurrence was described as a pneumothorax detected on a chest radiograph or computed tomography scan at presentation in our emergency room (ER) after treatment with 2P- or 3P-VATS. All patients received our standard postoperative medication regimen of non-opioid analgesics administered intravenously or orally. The patients received either metamizol natrium 1000 mg, paracetamol 1000 mg or ibuprofen 600 mg four times per day. In case of persistent pain using the standard pain medication regimen, we applied piritramide (opioid) 7.5 mg intravenously every 4–6 h on patient request. For each patient, the total opioid and non-opioid doses per stay were calculated and documented.

A week after discharge, the patients visited our outpatient clinic for postoperative control and follow-up. These visits were conducted at 3-month intervals for one year. A chest radiograph was taken at each visit. Patients were advised to visit our ER at any time they had symptoms related to recurrent pneumothorax (e.g., dyspnea, chest pain or cough). Recurrent pneumothorax was identified clinically in each case with a chest radiograph and a computed tomography scan of the lungs. For patients who recurred after VATS, repeated VATS or chest tube treatment was performed, depending on the underlying pulmonary disease, the patient’s clinical condition, as well as the patient’s choice. For long-term follow-up, patients were contacted and interviewed using a questionnaire.

The local ethics committee of the Heinrich-Heine University Clinic of Duesseldorf approved this study (study-no: 2020-1271).

### 2.1. Surgical Technique

Our specialized team of thoracic surgeons (A.S, A.R and WTK) performed all surgical procedures, consisting of a partial pleurectomy and bullectomy for ruptured bulla or blebs (for patients with extensive bullous disease, only the ruptured bleb/bulla and ultrathin bulla with high risk of rupture were resected). All the patients were treated under general anesthesia with a double-lumen tube intubation and single-lung ventilation. To open up the intercostal spaces, the patients were placed in a lateral position and the table was flexed up to 35°. Of note, the patients underwent either 2P-VATS or 3P-VATS initially, depending on the surgeon´s choice. However, in a few cases, the surgeons began surgery with 2P-VATS and then switched to 3P-VATS due to better accessibility and feasibility in these cases. 

### 2.2. Three-Port VATS 

**Figure 1:**
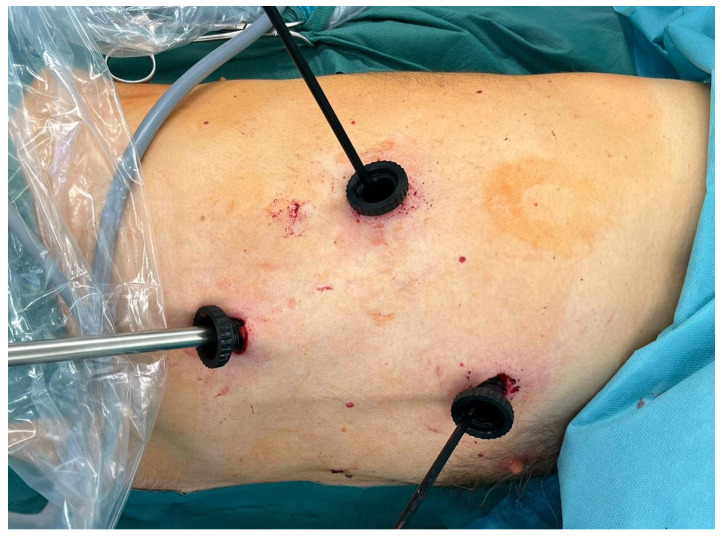
Trocar placement during 3P-VATS.

The 3P-VATS was performed as previously described [7]. Briefly, three 11 mm ports were placed at the level of the 5th intercostal space in the anterior axillary line and at the level of the 7th and 8th intercostal spaces in the mid- and posterior axillary lines, respectively. An endoscopic stapler (Autosuture GIA Universal; COVIDIEN^®^, Mansfield, MA, USA) was used for bullectomies. Partial pleurectomy was performed from the apex of the pleural cavity to the 7th or 8th intercostal space. To avoid vascular injury, the areas along the subclavian and internal mammary vessels were omitted (Figure 1).

### 2.3. Two-Port VATS 

**Figure 2:**
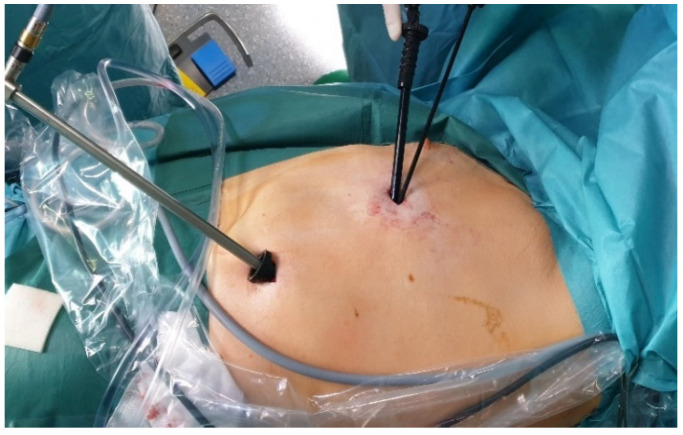
Trocar positioning for 2P-VATS.

The 2P-VATS was also performed as previously described [7]. In summary, two 11 mm ports were inserted at the level of the 5th and 8th intercostal space in the mid- and anterior axillary lines, respectively. For bullectomy, an endograsper and endoscopic stapling device were inserted side by side, without trocar guidance, via the incision in the 5th intercostal space [7] (Figure 2).

For both techniques, an underwater air leak test was performed and a 24 Fr chest tube was placed via the incision in the 5th intercostal space, to which a chest drainage system (Thopaz+, Medela AG, Baar, Switzerland) with a suction equivalent of −20 cm H2O was connected. During the postoperative course, the chest tube drain was removed when there were no clinical signs of air leaks, and when the daily drain output was less than 200 mL. After chest tube removal, a chest radiograph was taken to verify full expansion of the lung.

### 2.4. Statistical Analysis

All data were evaluated using the SPSS 25.0 software program (Statistical Package for Social Sciences; SPSS Inc., Chicago, IL, USA). Categorical variables were expressed as numbers and percentages, and continuous variables were presented as means. Fisher’s exact test was used to compare categorical data and the Mann–Whitney U test was applied for continuous data. Statistical significance was considered at *p* < 0.05.

## 3. Results

A total of 115 eligible patients were included in this study. Fifty-two patients, with a mean age of 67.7 years (range 41–87), underwent two-port VATS (2P-VATS), while 63 patients (mean age 68.6 years; range 44–87) were treated by three-port VATS (3P-VATS). The clinical characteristics (Table 1), such as mean age, gender, side of pneumothorax and COPD stage (I to IV), were similar in both groups. Between both groups, the BMI and initial size of the pneumothorax were significantly different (Table 1). Nine patients underwent 2P-VATS for other etiological causes (no COPD group: 7 patients had cavernous tuberculosis and 2 patients suffered from early stage I lung cancer (this patient underwent an anatomical lung resection)). Eight patients were treated with 3P-VATS for cavernous tuberculosis, one patient for early stage I lung cancer, and one patient for pneumocystis pneumonia. 

Regarding the surgical characteristics (Table 2), there was no significant difference in the number of resected lung segments between both groups, suggesting a lack of selection bias, based on the resected lung segments. The mean operation time (70.3 min vs. 91.4 min; *p* < 0.001) and the length of hospital stay (LOS) (10.7 days vs. 14.3 days; *p* = < 0.001) were significantly shorter for patients in the 2P-VATS group compared with patients in the 3P-VATS group. Additionally, patients who underwent 3P-VATS required a significantly higher total dose of opioid (41.6 mg vs. 24.5 mg; *p* < 0.001) and non-opioid (26.3 mg vs. 15.1 mg; *p* < 0.001) analgesics per stay, compared to patients following 2P-VATS. Interestingly, the total area of resected pleura, during pleurectomy, was significantly larger in the 3P-VATS group compared with the 2P-VATS group. We assume that the additional port access in the 3P-VATS group allowed for better feasibility of pleurectomy, due to the three-dimensional placement of the working trocars, compared to the 2P-VATS group, with limited two-dimensional placement of the trocars. Nine patients in the 3P-VATS group suffered a postoperative hemothorax, whereas this was the case in only three patients in the 2P-VATS group. We assumed that the large area of pleurectomy, following 3P-VATS, contributed to this high rate of hemothorax. Three patients in the 3P-VATS group required repeated VATS, due to hemothorax; the other six patients, and the three patients in the 2P-VATS group, were successfully treated conservatively. Similarly, all the patients with prolonged air leaks received conservative treatment until full recovery. During the clinical course, 5 patients suffered from acute pneumonia in the 2P-VATS group, compared to 9 patients in the 3P-VATS group. During a median follow-up period of 76.5 months (range 1–155 months), there was no significant difference in recurrence rates between the two groups (2P-VATS vs. 3P-VATS: 9.6% vs. 11.1%; *p* = 1.000).

## 4. Discussion

To date, reports on the outcomes following surgery for secondary spontaneous pneumothorax (SSP) are limited in the literature. Although surgery is associated with low rates of recurrence, high rates of morbidity and mortality after surgical treatment have been reported [5,6].

These high rates of morbidity and mortality are certainly not only impacted by the underlying pulmonary disease, but also by the surgical technique used. In the last decade, thoracic surgery has evolved from open thoracotomy to video-assisted thoracoscopic surgery (VATS), as the gold standard. While three-port VATS (3P-VATS) still remains the standard of care in most centers, due to its accessibility, recent surgical and technical developments are leading to a reduction in access ports [7]. While there are abundant reports on the surgical performance and benefits of limited port access, in terms of postoperative pain, paresthesia and length of hospital stay (LOS), for the treatment of primary spontaneous pneumothorax (PSP) [8,9,10,11,12,14], there is a lack of information on VATS for the treatment of SSP in such reports. Therefore, the aim of our study was to analyze and compare postoperative pain, in terms of the total dose of analgesics used per stay, and clinical outcome following 2P-VATS and 3P-VATS for SSP in our institution.

In this retrospective study, 52 patients underwent 2P-VATS, while 63 patients received 3P-VATS. The patients in the 2P-VATs group had a significantly lower total dose of analgesics administered per stay compared to the patients operated on with 3P-VATS, indicating less postoperative pain. Compared to some previously reported studies [8,9,10,11,12,14] that assessed postoperative pain using the visual analogue scale (VAS score), we used the total dose of non-opioids and opioids per patient as an objective surrogate for postoperative pain. In contrast to the VAS score, which displays a one-time measurement, including the patient´s psychological and emotional state, the quantification of applied analgesics allows pain assessment over a long period of time, independent of the patient´s pschyco-emotional state.

In addition, following 2P-VATS, the mean operation time and the LOS were significantly shorter. In terms of postoperative complications, the 2P-VATS patients had a low rate of hemothorax and pneumonia compared to the 3P-VATS patients (Table 2). We suggest that the high rate of hemothorax in the 3P-VATS group was related not only to the large area of pleurectomy resected during this procedure, but also to the additional port access. Similarly, we assume that the high rate of acute postoperative pneumonia in the 3P-VATS group was related to a higher postoperative pain level, which might have impaired breathing exercises during the first postoperative days. We believe that the high rate of postoperative complications also prolonged the LOS in the 3P-VATS group. To assess recurrence rates following 2P- and 3P-VATS, all the patients were followed-up for a mean period of 76.5 months (range 1–155 months). Interestingly, there was no significant difference in recurrence rates between both groups (2P-VATS 9.6% vs. 3P-VATS 11.1%; *p* = 1.000).

The power of our study is limited due to its retrospective design and the small number of patients included. To the best of our knowledge, this is the first study that analyses and compares postoperative pain and treatment outcome following 2P- and 3P-VATS for SSP patients. Our study demonstrates that the treatment of SSP by 2P-VATS is associated with less postoperative pain, low morbidity rates and earlier patient recovery compared to the conventional 3P-VATS. Nonetheless, this observation should be verified in a prospective trial with a larger number of patients.

## 5. Conclusions

According to our results, 2P-VATS is a suitable and safe surgical technique for the treatment of SSP. When compared to 3P-VATS, it is associated with less postoperative pain, lower morbidity rates and faster patient recovery. Therefore, 2P-VATS should be preferred for the surgical treatment of SSP.

## Figures and Tables

**Table 1:** Patient characteristics.

Variables	2P-VATS *n* = 52 (%)	3P-VATS *n* = 63 (%)	*p*-Value
Age (years)	67.7 (range 41–87)	68.6 (range 44–87)	0.808
Gender			
Male	35 (67.3)	32 (49.2)	
Female	17 (32.7)	32 (50.8)	0.060
Weight (kg)	66.1	64.1	0.223
Height (m)	1.72	1.73	0.297
BMI (kg/m^2^)	22.4	21.2	0.036 *
Collins (A + B + C) (cm)	8.6	10.8	0.029 *
COPD stage			
Gold I–II	22 (42.3)	23 (36.5)	0.568.
Gold III–IV	21 (40.4)	30 (47.6)	0.457
No COPD	9 (17.3)	10 (15.9)	1.000
Side of pneumothorax			
Right	34 (65.4)	47 (74.6)	
Left	18 (41.2)	16 (25.4)	0.310

Data are presented as means, numbers and percentages. COPD: chronic obstructive pulmonary disease, BMI: body mass index, 2P-VATS: two-port video-assisted thoracoscopic surgery, 3P-VATS: three-port video-assisted thoracoscopic surgery, LOS: length of hospital stay, Collins (A + B + C) = sum of the intrapleural distances (cm) according to the regression formula derived from Collins et al. [13]. * *p*-value < 0.05 indicates statistical significance.

**Table 2:** Surgical and postoperative characteristics.

Variables	2P-VATS *n* = 52 (%)	3P-VATS *n* = 63 (%)	*p-*Value
LOS (days)	10.7	14.3	<0.001 *
Opioid dosage/stay (mg)	24.5	41.6	<0.001 *
Non-opioid dosage/stay (g)	15.1	26.3	<0.001 *
Operation time (min)	70.3	91.4	<0.001 *
Length of air leak (days)	5.6	5.9	0.403
Area of pleurectomy (cm^2^)	17.2	32.1	0.006 *
Number of resected segments			
One-segment	41 (78.8)	50 (79.4)	0.934
Multi-segment	11 (21.2)	13 (20.6)	0.934
Postoperative complications			
Hemothorax	3 (5.8)	9 (14.3)	0.220
Acute pneumonia	5 (9.6)	9 (14.3)	0.571
Recurrence	5 (9.6)	7 (11.1)	1.000

Data are presented as means, numbers and percentages. 2P-VATS: two-port video-assisted thoracoscopic surgery, 3P-VATS: three-port video-assisted thoracoscopic surgery, LOS: length of hospital stay, min: minutes. * *p*-value < 0.05 indicates statistical significance.

## Data Availability

The data presented are included in this study; the corresponding author on request may provide additional data.
